# Lack of Association of Polymorphism Located Upstream of *ABCA1* (rs2472493), in *FNDC3B* (rs7636836), and Near *ANKRD55*–*MAP3K1* Genes (rs61275591) in Primary Open-Angle Glaucoma Patients of Saudi Origin

**DOI:** 10.3390/genes14030704

**Published:** 2023-03-13

**Authors:** Altaf A. Kondkar, Tahira Sultan, Taif A. Azad, Essam A. Osman, Faisal A. Almobarak, Glenn P. Lobo, Saleh A. Al-Obeidan

**Affiliations:** 1 Department of Ophthalmology, College of Medicine, King Saud University, Riyadh 11411, Saudi Arabia; tasayed@ksu.edu.sa (T.S.); mtanwar@ksu.edu.sa (T.A.A.); eosman@ksu.edu.sa (E.A.O.); falmobarak@ksu.edu.sa (F.A.A.); salobeidan@ksu.edu.sa (S.A.A.-O.); 2 Glaucoma Research Chair in Ophthalmology, College of Medicine, King Saud University, Riyadh 11411, Saudi Arabia; 3 King Saud University Medical City, King Saud University, Riyadh 11411, Saudi Arabia; 4 Department of Ophthalmology and Visual Neurosciences, University of Minnesota, Minneapolis, MN 55347, USA; lobo0023@umn.edu

**Keywords:** genetics, glaucoma, intraocular pressure, POAG, polymorphisms, rs2472493, rs7636836, rs61275591, Saudi

## Abstract

Polymorphisms rs2472493 near *ABCA1*, rs7636836 in *FNDC3B*, and rs61275591 near the *ANKRD55–MAP3K1* genes were previously reported to exhibit genome-wide significance in primary open-angle glaucoma (POAG). Since these polymorphisms have not been investigated in the Arab population of Saudi Arabia, we examined their association with POAG in a Saudi cohort. Genotyping was performed in 152 POAG cases and 246 controls using Taqman real-time assays and their associations with POAG and clinical markers, such as intraocular pressure, cup/disc ratio, and the number of antiglaucoma medications, were tested by statistical methods. There was no association observed between POAG and the minor allele frequencies of rs2472493[G], rs7636836[T], or rs61275591[A]. None of the genetic models such as co-dominant, dominant, recessive, over-dominant, and log-additive demonstrated any genotype link. The Rs2472493 genotype showed a modest association (*p* = 0.044) with the number of antiglaucoma medications in the POAG group, but no significant genotype effect on post hoc analysis. In addition, a G-T allelic haplotype of rs2472493 (*ABCA1*) and rs7636836 (*FNDC3B*) did show an over two-fold increased risk of POAG (odds ratio = 2.18), albeit non-significantly (*p* = 0.092). Similarly, no other allelic haplotype of the three variants showed any significant association with POAG. Our study did not replicate the genetic association of rs2472493 (*ABCA1*), rs763683 (*FNDC3B*), and rs61275591 (*ANKRD55*–*MAP3K1*) in POAG and related clinical phenotypes, suggesting that these polymorphisms are not associated with POAG in a Saudi cohort of Arab ethnicity. However, large population-based multicenter studies are needed to validate these results.

## 1. Introduction

Glaucoma is a heterogeneous group of eye diseases that affect more than 60 million individuals globally and is the second most common cause of irreversible blindness, including in the Saudi population [1]. Primary open-angle glaucoma (POAG) is the most common form of glaucoma with clinical features such as progressive death of retinal ganglion cells (RGC), damage to the optic nerve, and loss of vision [2]. Risk factors for POAG include aging, gender, increased intraocular pressure (IOP), family history, and African ancestry [3]. POAG is inherited as a complex trait; familial studies and genome-wide genetic association studies (GWAS) have led to strong evidence for the important contribution of genetic influence in the pathogenesis of POAG [4]. Several GWAS have identified significant genetic association in multiple genes and loci but with a varying effect on POAG outcomes [5], including the rs2472493 (chromosome 9, Position 106735669) located upstream of the ATP-binding cassette subfamily A (ABC1) member 1 (*ABCA1*); rs7636836 (chromosome 3, Position 173247819) in fibronectin type III domain containing 3B (*FNDC3B*); and rs61275591 (chromosome 5, Position 55811313) flanking the *ANKRD55*–*MAP3K1* intergenic loci [6,7].

ABCA1 and polymorphisms in this gene are recognized for their role in lipid metabolism [8,9]. Studies also support their contribution in neuroinflammatory and neurodegenerative conditions, RGC apoptosis, and plausibly a regulatory role in eye development [10,11,12]. Polymorphism rs2472493 is located in the 5′ upstream region near the *ABCA1* gene and has shown a significant association with POAG and IOP in the European and Asian populations [6]. Similar associations were reported in an Australian cohort [10] and POAG patients from southern China [13], supporting a crucial role of *ABCA1* genetic polymorphism in POAG pathogenesis. Similarly, another genome-wide study reported two novel polymorphisms, rs7636836 and rs61275591, to be associated with the Japanese POAG cases [7]. Rs7636836 in *FNDC3B* encoding an extracellular matrix protein may contribute to the regulation of the TGFβ pathway which is strongly implicated in glaucoma pathogenesis [14,15]. The presence of a genetic variant could potentially disrupt these signaling pathways and contribute to the development of glaucoma. Meanwhile, rs61275591 is flanked in between two genes, *ANKRD55* and *MAP3K1*, of unknown significance. *ABCA1*, *FNDC3B*, and *ANKRD55*–*MAP3K1* are all highly expressed in the tissues relevant to glaucoma, supporting their plausible role in ocular development and glaucoma-related phenotypes [7,10]. Moreover, these polymorphisms might alter certain regulatory motifs and histones, and have been associated with cis-expression quantitative trait loci (eQTLs) as predicted using the NIH SNP function prediction (https://snpinfo.niehs.nih.gov/snpinfo/snpfunc.html, accessed on 11 December 2022) and HaploReg v4.1 web tools highlighting their plausible regulatory function (Supplementary Table S1). 

Glaucoma is a prevalent disease in the Arab population, with some studies reporting a higher prevalence rate compared to other populations [1]. The Middle East is also known for its high rate of consanguinity, which suggests that genetics might play a role in certain subsets of this population. This makes it crucial to investigate the genetic basis of glaucoma in Arab populations, as genetic factors may be contributing to the high prevalence of the disease. However, despite the need for research, there is currently a lack of consistent data on the possible causative genes or variants involved in glaucoma in this population [16,17,18,19]. Therefore, genetic association studies are needed to provide a better understanding of the genetic factors underlying glaucoma in the Arab population. Moreover, genome-wide studies identifying genetic polymorphism(s) or loci to be associated with POAG have been mostly performed in Caucasians, Europeans, or Asians and need replication in other ethnicities. Using a case-control candidate gene approach we investigated the association of polymorphisms rs2472493 upstream of *ABCA1*, rs7636836 in *FNDC3B*, and rs61275591 near *ANKRD55*–*MAP3K1* genes with POAG and its related phenotypes in a Saudi cohort.

## 2. Materials and Methods

### 2.1. Design and Study Population

A retrospective case-control study was performed. POAG patients and controls were recruited at King Abdulaziz University Hospital, Riyadh, Saudi Arabia as described in detail elsewhere [20]. Briefly, POAG patients showed the presence of (1) glaucomatous changes at the optic disk or retinal nerve fiber changes (such as narrowing or absence of neuroretinal rim, cup-to-disc ratio exceeding 0.7, an inter-eye asymmetry of >0.2, and/or notching); (2) corresponding visual field defects typical of glaucoma; (3) bilaterally open angles; (4) late-onset; (5) high IOP (≥21 mm Hg) in one or both eyes before treatment; (6) and no secondary causes of glaucomatous optic neuropathy with identifiable causes such as exfoliative glaucoma, angle-closure, pigmentary glaucoma, post-traumatic, infectious or inflammatory glaucoma (e.g., uveitis), or post-surgical and post-medication glaucoma (e.g., after corticosteroids). The controls were of the same ethnicity, with normal IOP (<21 mm Hg with no medication), no glaucoma, normal optic disc (cup/disc ratio <0.5), absence of a family history of glaucoma, and aged ≥40 years. Individuals who did not wish to participate in the study were excluded.

### 2.2. Genotyping of rs2472493, rs7636836, and rs61275591 Polymorphisms

DNA extracted from EDTA blood were genotyped for the rs2472493 (A > G), rs7636836 (C > T), and rs61275591 (G > A) polymorphisms using commercially available TaqMan^®^ assays, C__16235609_10, C_189412462_10, and C__88653935_10 (catalog number: 4351379; Applied Biosystems Inc., Foster City, CA, USA) on ABI-7500 real-time PCR (Applied Biosystems) as recommended by the manufacturer and described previously [21]. 

### 2.3. Statistics

SPSS version 22 (IBM Inc., Chicago, IL, USA), Stat View software version 5.0 (SAS Institute, Cary, NC, USA), SNPStats (https://www.snpstats.net/start.htm, accessed on 11 December 2022), and SHEsis (http://analysis.bio-x.cn/myAnalysis.php, accessed on 11 December 2022) were used for statistical analysis. Normality distribution was tested using the Kolmogorov–Smirnov test. Non-parametric methods such as the Mann–Whitney U test were used to examine age differences and the Kruskal–Wallis test was used to evaluate the effect of genotypes on clinical phenotypes. Hardy–Weinberg Equilibrium (HWE), gender differences, and genetic association testing were performed using Pearson’s chi-square analysis or Fisher’s exact test. The effects of multiple risk factors such as age, sex, and genotypes on POAG were examined by logistic regression analysis. The linkage disequilibrium (LD) and combined allelic (haplotype) effect of polymorphisms were examined by SHEsis. Power calculation was performed using PS program version 3.1.2 (https://vbiostatps.app.vumc.org/ps/, accessed on 11 December 2022). A *p* < 0.05 (2-tailed) was considered significant. Bonferroni’s correction *p*-value (0.05/5 = 0.01) for multiple testing was considered when applicable.

## 3. Results

### 3.1. Demographic and Minor Allele Frequency Distribution

The age, gender, and minor allele frequency (MAF) distribution of participants included in the study is shown in Table 1. As shown, age and gender showed no significant difference between POAG cases and controls. 

Overall, 246 controls and 152 POAG cases were included in the study. Of the total number of 398 samples genotyped in our cohort, the success rate of genotyping was 99%, 100%, and 99% for rs2472493, rs7636836, and rs61275591 polymorphisms, respectively. Five DNA samples—one control and one patient for rs2472493, and three controls for rs61275591—failed to amplify. The samples with missing genotypes were not included in their respective single and combined allele/genotype analysis. The allelic distribution showed no significant deviation from HWE (*p* > 0.05). As shown in Table 1, there was no significant difference in age and gender distribution in cases and controls. In addition, none of the three polymorphisms showed any significant overall or gender-specific association with POAG (Table 1).

### 3.2. Genotype Association Analysis with POAG

Since POAG does not show a clear inheritance pattern, genotype association analysis was conducted using multiple genetic models with SNPStats software. Polymorphism rs2472493, rs7636836, and rs61275591 genotypes showed no significant association with POAG in univariate, and age- and sex-adjusted analysis (Table 2, Table 3 and Table 4). Likewise, the genotype analysis according to gender categorization was also not associated with POAG in any genetic models (Table 2, Table 3 and Table 4).

### 3.3. Combined Genotype, Linkage, and Haplotype Analysis

The three polymorphisms were tested for LD and subjected to haplotype analysis using the SHEsis online version. The standardized LD coefficient D’ and r^2^ values between rs2472493, rs7636836, and rs61275591 indicated that these polymorphisms are not in LD (Supplementary Figure S1). In addition, the haplotype distribution did not show any significant association with POAG (overall *X*^2^ = 10.43, df = 6, *p* = 0.107) (Table 5). However, a G-T haplotype of rs2472493 of *ABCA1* and rs7636836 of *FNDC3B* did increase the risk of POAG by more than twofold (OR = 2.18, 95% and CI = 0.86–5.51) albeit non-significantly (*p* = 0.092). In addition, combined genotype analysis of AA-CC-GG of rs2472493, rs7636836, and rs61275591, respectively, versus other genotypes also did not show any significant association with POAG (OR = 1.27, 95% CI = (0.79–2.04), *p* = 0.345).

### 3.4. Binary Logistic Regression Analysis and Genotype Influence on Clinical Markers

**Figure 1:**
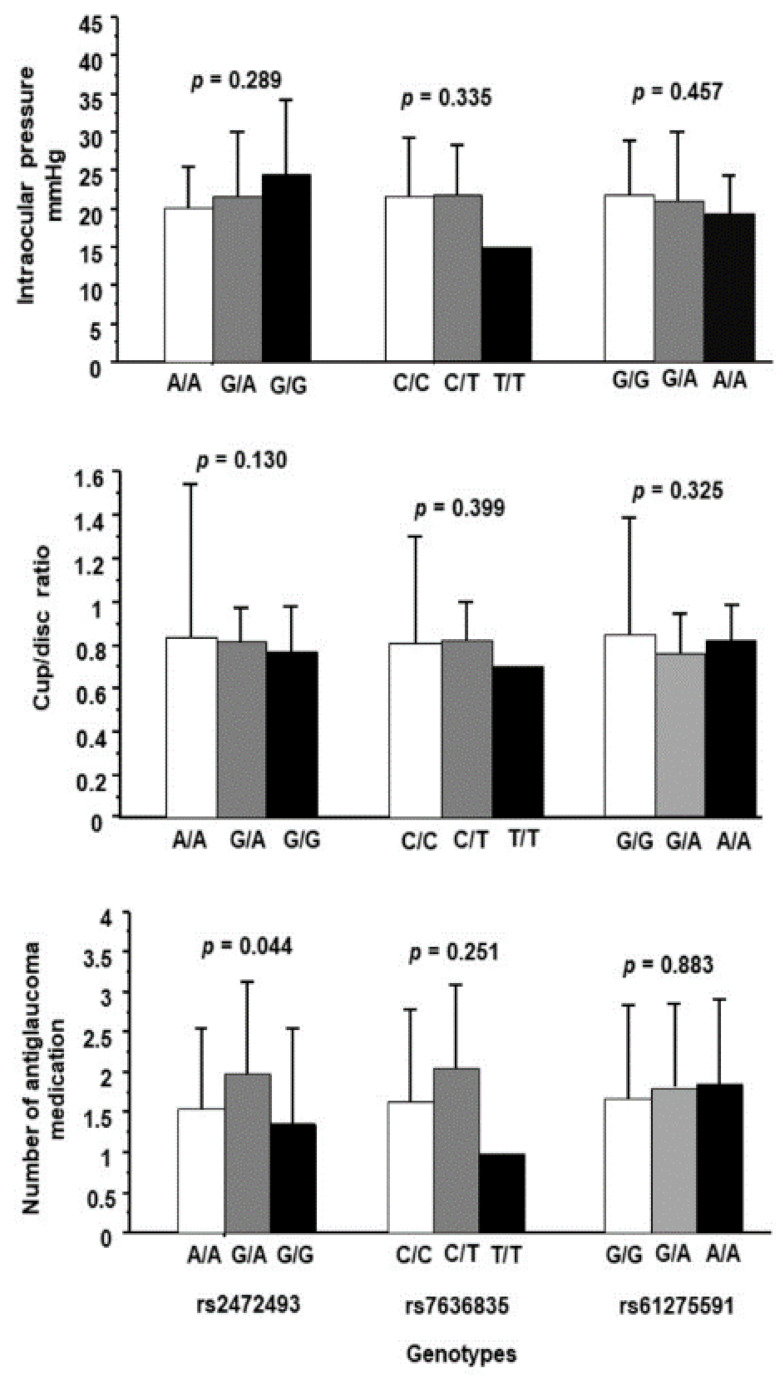
Genotype effects of polymorphisms rs2472493 (*ABCA1*), rs7636836 (*FNDC3B*), and rs61275591 *(ANKRD55*–*MAP3K1*) on intraocular pressure, cup/disc ratio, and the number of antiglaucoma medications in primary open-angle glaucoma (POAG) patients.

To determine the impact of several risk factors, including age, sex, and the genotypes of rs2472493, rs7636836, and rs61275591 on the outcome of POAG, a binary logistic regression analysis was conducted. This analysis showed that the risk of POAG was not significantly influenced by any of these criteria. (Table 6). Moreover, these polymorphisms showed no significant genotype influence on clinical markers of disease severity such as IOP, cup/disc ratio, and the number of anti-glaucoma drugs (Figure 1). Although the rs2472493 polymorphism near *ABCA1* did show a moderately significant genotype effect with the number of antiglaucoma medications of (*p* = 0.044) in POAG, the post hoc analysis did not show any significant genotype effect. In addition, further analysis of rs2472493 in dominant and recessive models did not show any significant genotype association with the number of antiglaucoma medications variable (*p* = 0.154 and *p* = 0.142, respectively).

## 4. Discussion

The polygenic nature, high probability of inheritance, and race-specific prevalence to POAG suggests a risk of genetic predisposition to the disease [5,16,22]. Although polymorphisms in a number of genes or loci have linked with POAG, the exact molecular contribution of these genes toward POAG development and/or progression is still unclear [5,16]. Likewise, the genetic basis of POAG in the middle-eastern Arab population is not clear and remains to be identified [19]. Thus, we investigated three previously reported polymorphisms that had been associated with POAG and/or increased IOP [6,7]. However, we observed no association of these polymorphisms with POAG and other related clinical markers. 

Rs2472493[G] near *ABCA1* had a MAF of 0.38 in our POAG cases and was not significantly different compared to controls (0.39). As compared to other ethnicities, the MAFs were found to be similar to the POAG population of Hispanics (0.37), and African American (0.35) [23], but lower than the POAG population of non-Hispanics (0.42), East Asians (0.56) [23], and Europeans (0.51) [10], exhibiting ethnic variations.

Hysi et al., using a genome-wide meta-analysis study, first reported an association of polymorphism rs2472493 near *ABCA1* with POAG in a multi-ethnic population of European and Asian ancestry [6]. The association was successfully replicated in POAG patients in Australia [10] and southern China [13]. However, our study failed to replicate these findings in POAG patients of Saudi origin. Similarly, no associations of *ABCA1* gene polymorphism(s) have been reported among a PACG population of Han Chinese [24], in a Brazilian POAG cohort [25], and in Arab POAG and congenital glaucoma patients from Jordan [17]. This supports our finding that polymorphism rs2472493 in *ABCA1* may not have a major role in the POAG pathogenesis of Saudi Arab ethnicity. 

There are enough data to implicate the role of ABCA1 in eye diseases, including glaucoma. Expression of ABCA1 has been confirmed in several ocular tissues relevant to glaucoma [13]. An in vivo study has demonstrated the role of ABCA1 in the inhibition of ocular inflammation by activating liver X-receptor in autoimmune uveitis [26]. In another glaucoma model, ABCA1 was related to RGC apoptosis [27]. Likewise, a strong linkage has been reported between rs2472493 and rs2472494 near *ABCA1* that may modify the regulatory binding motif sequence of PAX6, suggesting a plausible regulatory role in ocular development [10]. However, the exact molecular mechanism(s) are still unknown. This is in contrast to our negative findings which indicate that polymorphism rs2472493 located near *ABCA1* might not be a major player in POAG pathogenesis in Middle Eastern Saudi Arab patients. There could be several possible reasons for the noted absence of this association in the Saudi cohort. The absence of an association between the rs2472493 polymorphism in *ABCA1* and POAG in Saudi Arabs could be due to several factors. Genetic differences may affect the expression of certain genes and their association with diseases, and this could be a possible reason for the lack of association in Saudi Arabs. In addition, a relatively small number of samples investigated in this study may not have enough statistical power to detect a significant association. Environmental factors, such as diet or lifestyle, may also play a role in influencing the outcome. Moreover, clinical differences or variations in POAG patients could also be a factor that could contribute to the lack of association in Saudi Arab populations. However, the association of other polymorphisms in this gene with POAG cannot be completely ruled out and requires further investigation. 

The expression of *FNDC3B* has been demonstrated in all the ocular tissues involved in glaucoma [7,28]. *FNDC3B* plays a regulatory role in the adipocyte formation [29] and encodes proteins involved in cell adhesion, and TGFβ and Wnt/β-catenin signaling pathways [30,31,32]. These processes and pathways play a critical role in glaucoma pathophysiology and could be the plausible mechanism(s) by which rs7636836 in *FNDC3B* might influence the risk of POAG [14,33,34,35]. In a large population-based study of POAG patients in Japan, Shiga et al. reported the genome-wide association of polymorphism rs7636836 in the *FNDC3B* gene [7]. However, these findings were not replicated in Singapore Chinese, European, and African populations [7]. The observed MAFs of rs7636836[T] in *FNDC3B* in our POAG patients and controls were 0.05 and 0.07, respectively. The MAF was comparable to the European POAG population (0.06) but lower than those observed in POAG patients from Japan (0.40) and Chinese patients in Singapore (0.21) [7]. No significant allelic and genotype association was seen between rs7636836 in *FNDC3B* and our POAG cohort.

In addition to rs7636836, the rs61275591 locus was also one of the novel loci associated with POAG in the Japanese population in the same study by Shiga et al. [7]. The MAFs reported were 0.27 in the Japanese, 0.31 in the Singaporean Chinese, 0.08 in the European, 0.07 in the African American, and 0.06 in the African population as compared to 0.17 observed in our Saudi cohort, which is lower than the East Asians but higher than the Africans. Rs61275591 was not associated with POAG in our cohort as was reported in the Singaporean Chinese, European, and African populations [7]. The rs61275591 locus is mapped between *ANKRD55* and *MAP3K1* genes. *ANKRD55* encodes ankyrin repeat domain-containing protein 55, which mediates protein–protein interactions and may have a role in autoimmune diseases [36], whereas MAP3K1, which is also known as MEK kinase 1, is a serine/threonine kinase that belongs to the mitogen-activated protein kinases (MAP3K) family. MAP3K1 helps regulate signaling pathways that control various processes in the body, including sex determination, cell development, differentiation, migration, survival, and apoptosis. Genomic alterations in MAP3K1 have been reported in diverse cancer types and the Swyer syndrome [37,38]. However, the possible relevance of *ANKRD55* and *MAP3K1* in glaucoma is unknown. 

Epistatic interactions are known to play a critical role in the pathogenesis of polygenic multifactorial diseases highlighting the plausible role of genetic background and interplay in disease outcome and progression [39,40,41]. Since glaucoma is also polygenic in nature with no clear pattern of inheritance, there may exist similar mechanism(s) that might modulate the disease processes [42]. Thus, to examine any synergistic effect, we conducted a combined allelic and genotype analysis of rs2472493, rs7636836, and rs61275591 in POAG. However, none of the genotype and allelic (haplotype) combinations showed any significant association with POAG. Nonetheless, the possible effect(s) of other polymorphism(s) in LD or other epistatic interactions cannot be ruled out.

The genetic variants of *ABCA1* and *FNDC3B* have also been linked with increased IOP [6]. ABCA1 was demonstrated to regulate IOP via the CAV-1/NO/eNOS pathways [43]. However, all three polymorphisms investigated in this study showed no genotype effect on the clinical markers of disease severity in our cohort. Similarly, rs2472494 (in strong LD with rs2472493), rs7636836, and rs61275591 in a few of the glaucoma loci investigated by Chai et al. were recently reported to show no association with optic disc parameters [44].

Therefore, our findings suggest that rs2472493 upstream of *ABCA1*, rs7636836 in *FNDC3B*, and rs61275591 near *ANKRD55*–*MAP3K1* may not play a significant role in POAG in Arabs of Saudi origin. However, to rule out the role of other polymorphisms in these genes, the epistatic, and gene-environment interaction need further investigation. Moreover, the relatively small sample size used in this study further warrants the need for replication in a well-designed, multicenter, population-based study to validate these results. The calculated power of our study was >0.9 per allele for the rs2472493 (*ABCA1*) and rs61275591 (*ANKRD55*–*MAP3K1*) polymorphisms but was underpowered (0.68 per allele) for the rs7636836 (*FNDC3B*) polymorphism to detect an odds of 2.0 and α = 0.05 but the sample size needs to be much larger to detect an effect size of 1.5 or less, as is commonly reported in polygenic case-control studies. 

We conclude that the polymorphisms rs2472493 upstream of *ABCA1*, rs7636836 in *FNDC3B*, and rs61275591 near the *ANKRD55* and *MAP3K1* genes are not associated with POAG in Middle Eastern Arabs of Saudi origin. However, the results require further confirmation in different regions and in much larger samples. 

## Figures and Tables

**Table 1:** Demographic characteristics and distribution of minor allele frequency of study participants.

Characteristics	Controls (n = 246)	POAG (n = 152)	Odds Ratio (95% Confidence Interval)	*p*-Value
Age in years (SD)	59.5 (7.2)	60.9 (10.5)	-	0.112
Male/Female, n	132/114	84/68	0.81 (1.06–0.06)	0.751
Minor Allele Frequency				
rs2472493[G]				
Total	0.39	0.38	0.95 (0.71–1.26)	0.700
Men	0.37	0.37	1.01 (0.69–1.48)	0.970
Women	0.42	0.39	0.88 (0.57–1.35)	0.560
rs7636836[T]				
Total	0.05	0.07	1.34 (0.74–2.41)	0.330
Men	0.04	0.07	1.61 (0.68–3.80)	0.273
Women	0.06	0.07	1.14 (0.52–2.51)	0.750
rs61275591[A]				
Total	0.17	0.18	1.09 (0.75–1.59]	0.629
Men	0.04	0.07	1.61 (0.68–3.80)	0.273
Women	0.06	0.07	1.14 (0.52–2.51)	0.750

**Table 2:** Association analysis of rs2472493 polymorphism upstream of ABCA1 with primary open-angle glaucoma.

Group	Genetic Model ^1^	Genotype	Controls n (%)	Cases n (%)	OR (95% CI)	*p*-Value ^2^	*p*-Value ^2,3^
Overall	Co-dominant	A/A	97 (39.6)	59 (39.1)	1.00	0.610	0.650
		G/A	104 (42.5)	70 (46.4)	1.11 (0.71–1.72)		
		G/G	44 (18.0)	22 (14.6%)	0.82 (0.45–1.51)		
	Dominant	A/A	97 (39.6)	59 (39.1)	1.00	0.920	0.960
		G/A-G/G	148 (60.4)	92 (60.9)	1.02 (0.67–1.55)		
	Recessive	A/A-G/A	201 (82.0)	129 (85.4)	1.00	0.380	0.380
		G/G	44 (18.0)	22 (14.6)	0.78 (0.45–1.36)		
	Over-dominant	A/A-G/G	141 (57.5)	81 (53.6)	1.00	0.450	0.540
		G/A	104 (42.5)	70 (46.4)	1.17 (0.78–1.76)		
	Log-additive	---	---	---	0.95 (0.71–1.26)	0.700	0.620
Men	Co-dominant	A/A	56 (42.4)	35 (41.7)	1.00	0.980	0.990
		G/A	55 (41.7)	36 (42.9)	1.05 (0.58–1.90)		
		G/G	21 (15.9)	13 (15.5)	0.99 (0.44–2.23)		
	Dominant	A/A	56 (42.4)	35 (41.7)	1.00	0.910	0.990
		G/A-G/G	76 (57.6)	49 (58.3)	1.03 (0.59–1.80)		
	Recessive	A/A-G/A	111 (84.1)	71 (84.5)	1.00	0.930	0.880
		G/G	21 (15.9)	13 (15.5)	0.97 (0.46–2.06)		
	Over-dominant	A/A-G/G	77 (58.3)	48 (57.1)	1.00	0.860	0.920
		G/A	55 (41.7)	36 (42.9)	1.05 (0.60–1.83)		
	Log-additive	---	---	---	1.01 (0.69–1.48)	0.970	0.930
Women	Co-dominant	A/A	41 (36.3)	24 (35.8)	1.00	0.430	0.530
		G/A	49 (43.4)	34 (50.8)	1.19 (0.61–2.31)		
		G/G	23 (20.4)	9 (13.4)	0.67 (0.27–1.68)		
	Dominant	A/A	41 (36.3)	24 (35.8)	1.00	0.950	0.970
		G/A-G/G	72 (63.7)	43 (64.2)	1.02 (0.54–1.91)		
	Recessive	A/A-G/A	90 (79.7)	58 (86.6)	1.00	0.230	0.280
		G/G	23 (20.4)	9 (13.4)	0.61 (0.26–1.40)		
	Over-dominant	A/A-G/G	64 (56.6)	33 (49.2)	1.00	0.340	0.430
		G/A	49 (43.4)	34 (50.8)	1.35 (0.73–2.47)		
	Log-additive	---	---	---	0.88 (0.57–1.35)	0.560	0.550

^1^ Tested by SNPStats; ^2^ chi-square analysis; ^3^ adjusted for age and sex. Bonferroni corrected *p*-value is 0.01. OR, odds ratio; CI, confidence interval.

**Table 3:** Association analysis of rs7636836 polymorphism in *FNDC3B* with primary open-angle glaucoma.

Group	Genetic Model ^1^	Genotype	Controls n (%)	Cases n (%)	OR (95% CI)	*p*-Value ^2^	*p*-Value ^2,3^
Overall	Co-dominant	C/C	224 (91.1)	133 (87.5)	1.00	0.480	0.480
		C/T	20 (8.1)	18 (11.8)	1.52 (0.77–2.97)		
		T/T	2 (0.8)	1 (0.7)	0.84 (0.08–9.38)		
	Dominant	C/C	224 (91.1)	133 (87.5)	1.00	0.260	0.250
		C/T-T/T	22 (8.9)	19 (12.5)	1.45 (0.76–2.79)		
	Recessive	C/C-C/T	244 (99.2)	151 (99.3)	1.00	0.860	0.930
		T/T	2 (0.8)	1 (0.7)	0.81 (0.07–8.99)		
	Over-dominant	C/C-T/T	226 (91.9)	134 (88.2)	1.00	0.230	0.220
		C/T	20 (8.1)	18 (11.8)	1.52 (0.78–2.97)		
	Log-additive	---	---	---	1.34 (0.74–2.41)	0.330	0.310
Men	--	C/C	121 (91.7)	73 (86.9)	1.00	0.260	0.250
		C/T	11 (8.3)	11 (13.1)	1.66 (0.68–4.02)		
		T/T	0 (0)	0 (0)	-		
Women	Co-dominant	C/C	103 (90.3)	60 (88.2)	1.00	0.850	0.900
		C/T	9 (7.9)	7 (10.3)	1.34 (0.47–3.77)		
		T/T	2 (1.8)	1 (1.5)	0.86 (0.08–9.67)		
	Dominant	C/C	103 (90.3)	60 (88.2)	1.00	0.650	0.680
		C/T-T/T	11 (9.7)	8 (11.8)	1.25 (0.48–3.28)		
	Recessive	C/C-C/T	112 (98.2)	67 (98.5)	1.00	0.880	0.970
		T/T	2 (1.8)	1 (1.5)	0.84 (0.07–9.39)		
	Over-dominant	C/C-T/T	105 (92.1)	61 (89.7)	1.00	0.580	0.640
		C/T	9 (7.9)	7 (10.3)	1.34 (0.47–3.78)		
	Log-additive	---	---	---	1.14 (0.52–2.51)	0.750	0.740

^1^ Tested by SNPStats; ^2^ chi-square analysis; ^3^ adjusted for age and sex. Bonferroni corrected p-value is 0.01. OR, odds ratio; CI, confidence interval.

**Table 4:** Association analysis of rs61275591 polymorphism near *ANKRD55*–*MAP3K1* genes with primary open-angle glaucoma.

Group	Genetic Model ^1^	Genotype	Controls n (%)	Cases n (%)	OR (95% CI)	*p*-Value ^2^	*p*-Value ^2,3^
Overall	Co-dominant	G/G	167 (68.7)	101 (66.5)	1.00	0.890	0.860
		A/G	69 (28.4)	46 (30.3)	1.10 (0.70–1.72)		
		A/A	7 (2.9)	5 (3.3)	1.18 (0.37–3.82)		
	Dominant	G/G	167 (68.7)	101 (66.5)	1.00	0.640	0.600
		A/G-A/A	76 (31.3)	51 (33.5)	1.11 (0.72–1.71)		
	Recessive	G/G-A/G	236 (97.1)	147 (96.7)	1.00	0.820	0.740
		A/A	7 (2.9)	5 (3.3)	1.15 (0.36–3.68)		
	Over-dominant	G/G-A/A	174 (71.6)	106 (69.7)	1.00	0.690	0.680
		A/G	69 (28.4)	46 (30.3)	1.09 (0.70–1.71)		
	Log-additive	---	---	---	1.10 (0.75–1.60)	0.630	0.580
Men	Co-dominant	G/G	90 (69.8)	56 (66.7)	1.00	0.790	0.730
		A/G	35 (27.1)	24 (28.6)	1.10 (0.59–2.04)		
		A/A	4 (3.1)	4 (4.8)	1.61 (0.39–6.69)		
	Dominant	G/G	90 (69.8)	56 (66.7)	1.00	0.630	0.590
		A/G-A/A	39 (30.2)	28 (33.3)	1.15 (0.64–2.08)		
	Recessive	G/G-A/G	125 (96.9)	80 (95.2)	1.00	0.540	0.470
		A/A	4 (3.1)	4 (4.8)	1.56 (0.38–6.43)		
	Over-dominant	G/G-A/A	94 (72.9)	60 (71.4)	1.00	0.820	0.80
		A/G	35 (27.1)	24 (28.6)	1.07 (0.58–1.98)		
	Log-additive	---	---	---	1.17 (0.71–1.92)	0.540	0.480
Women	Co-dominant	G/G	77 (67.5)	45 (66.2)	1.00	0.830	0.840
		A/G	34 (29.8)	22 (32.4)	1.11 (0.58–2.12)		
		A/A	3 (2.6)	1 (1.5)	0.57 (0.06–5.65)		
	Dominant	G/G	77 (67.5)	45 (66.2)	1.00	0.850	0.920
		A/G-A/A	37 (32.5)	23 (33.8)	1.06 (0.56–2.01)		
	Recessive	G/G-A/G	111 (97.4)	67 (98.5)	1.00	0.590	0.590
		A/A	3 (2.6)	1 (1.5)	0.55 (0.06–5.42)		
	Over-dominant	G/G-A/A	80 (70.2)	46 (67.7)	1.00	0.720	0.780
		A/G	34 (29.8)	22 (32.4)	1.13 (0.59–2.15)		
	Log-additive	---	---	---	1.01 (0.57–1.79)	0.980	0.960

^1^ Tested by SNPStats; ^2^ chi-square analysis; ^3^ adjusted for age and sex. Bonferroni corrected p-value is 0.01. OR, odds ratio; CI, confidence interval.

**Table 5:** Combined allelic effect of rs2472493 (*ABCA1*), rs7636836 (*FNDC3B*), and rs61275591 (*ANDPT55*-*MAP3K1*) variants in primary open-angle glaucoma.

Allele Combination ^1^	POAG Frequency	Controls Frequency	Fisher’s *p* ^2^	Odds Ratio (95% Confidence Interval)
A-C-G	0.495	0.505	0.770	0.95 [0.71~1.28]
A-C-A	0.101	0.073	0.180	1.41 [0.84~2.36]
A-T-G	0.030	0.032	0.887	0.94 [0.40~2.17]
A-T-A	0.001	0.002	-	-
G-C-G	0.268	0.276	0.786	0.95 [0.69~1.32]
G-C-A	0.069	0.096	0.186	0.69 [0.40~1.19]
G-T-G	0.024	0.016	0.414	1.53 [0.54~4.31]
G-T-A	0.013	0.000	-	-

^1^ Tested by SHEsis in the order of rs247243, rs7636836, and rs61275591; ^2^ Uncorrected *p*-value. Global *X*^2^ = 10.43, df = 6 (frequency < 0.01 not considered), Fisher’s *p*-value is 0.107. POAG, primary open-angle glaucoma.

**Table 6:** Binary logistic regression analysis to determine the effect of age, sex, rs2472493, rs7636836, and rs61275591 genotypes on the risk of disease outcome.

Group Variables	B	S.E.	Wald	*p*-Value	Odds Ratio (95% Confidence Interval)
Age	0.020	0.012	2.475	0.116	1.02 (0.99–1.04)
Sex	0.018	0.213	0.007	0.932	1.01 (0.67–1.54)
Rs2472493			0.988	0.610	-
G/A	0.053	0.233	0.052	0.820	1.05 (0.66–1.66)
G/G	−0.255	0.318	0.641	0.424	0.77 (0.41–1.44)
Rs7636836			1.709	0.426	-
C/T	0.451	0.346	1.700	0.192	1.57 (0.79–3.09)
T/T	−0.070	1.241	0.003	0.955	0.93 (0.08–10.6)
Rs61275591			0.349	0.840	-
G/A	0.078	0.235	0.109	0.741	1.08 (0.68–1.71)
A/A	0.320	0.608	0.278	0.598	1.37 (0.41–4.53)
Constant	−1.752	0.770	5.171	0.023	0.17

## Data Availability

The data supporting the conclusions of this article are all presented within the report.

## Supplementary Materials

The following supporting information can be downloaded at: https://www.mdpi.com/article/10.3390/genes14030704/s1, Figure S1: Linkage disequilibrium test between rs2472493 (Site1), rs7636836 (Site 2) and rs61275591 (Site3) using SHEsis online version (http://analysis.bio-x.cn/, accessed on 11 December 2022); Table S1: Details of the genomic region of the polymorphisms location, frequency and its predicted neighboring features such as transcription binding sites, altered regulatory elements, eQTLs, miRNA binding sites, and conservation among species obtained using NIH SNPinfo(https://snpinfo.niehs.nih.gov/snpinfo/snpfunc.html, accessed on 11 December 2022) and HaploRegv4.1 web tools.
