# Supplementary material for: Lack of Association of Polymorphism Located Upstream of ABCA1 (rs2472493), in FNDC3B (rs7636836), and Near ANKRD55–MAP3K1 Genes (rs61275591) in Primary Open-Angle Glaucoma Patients of Saudi Origin
Source: Genes (Basel). 2023 Mar 13;14(3):704. doi: 10.3390/genes14030704 (PMC10048255; doi:10.3390/genes14030704)
Supplement: Supplementary file 1 [file genes-14-00704-s001.zip › genes-2226825-supplementary.pdf]

Supplementary Table S1: Details of the genomic region of the polymorphisms location, frequency and its predicted neighboring features such as transcription binding sites, altered regulatory elements, eQTLs, miRNA binding sites, and conservation among species obtained using NIH SNPinfo (https://snpinfo.niehs.nih.gov/snpinfo/snpfunc.html) and HaploReg v4.1 web tools.

SNPID	rs2472493	rs7636836	rs61275591
Chromosome	9	3	5
Position	106735669	173247819	55811313
Allele	G/A	C/T	A/G
Nearby Gene(s)	ABCA1||SLC44A1	LOC100130245||FNDC3B	LOC345645||LOC441073
Frequency	G=0.3959, A=0.6041 (GnomAD)	C=0.9041, T=0.0959 (GnomAD)	G=0.9207, A=0.07933 (GnomAD)
	G=0.3872, A=0.6128 (1000Genomes)	C=0.871, T=0.129 (1000Genomes)	G=0.856, A=0.144 (1000Genomes)
	G=0.4009, A=0.5991 (TOPMED)	C=0.9024, T=0.09756 (TOPMED)	G=0.911, A=0.08904 (TOPMED)
Promoter histone marks	--	2 tissues	5 tissues
Enhancer histone marks	4 tissues	10 tissues	14 tissues
DNAse	--	--	9 tissues
Proteins bound	--	--	GATA2
Regulatory Motifs changed	Foxj2, Msx1, Pou2f2, Pou3f3, Six5_disc3	Mef2, Sox, E2f3	AP-2, Zic
NHGRI/EBI GWAS hits	2 hits	2 hits	--
Selected eQTL hits	22 hits	22 hits	--
miRNA (miRanda)	--	--	--
Splicing(ESE or ESS)	--	--	--
Conservation	0	0.682	Not available

D':	Site2	Site3
Site1	0.029	0.209
Site2	-	0.329

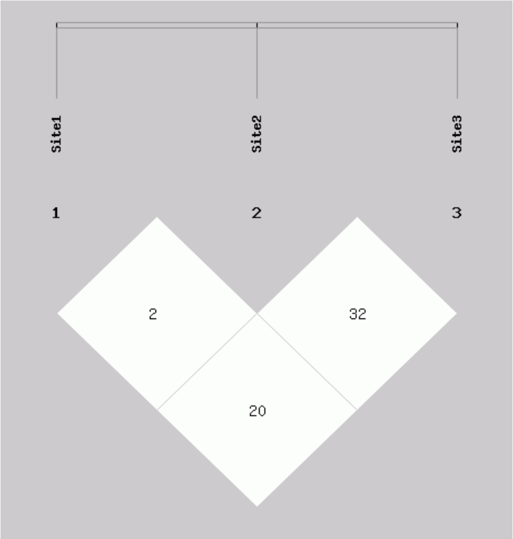

r2:	Site2	Site3
Site1	0.000	0.015
Site2	-	0.001

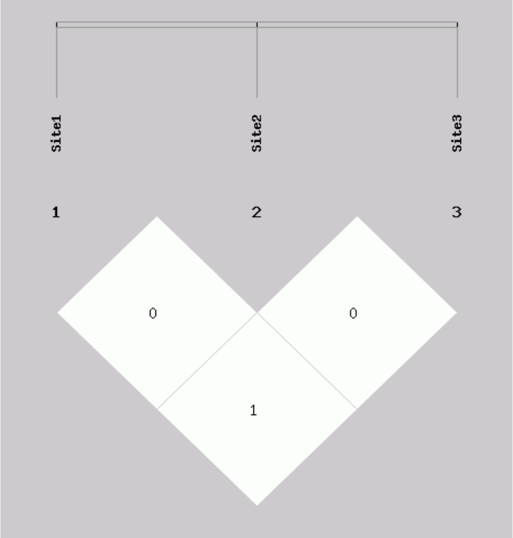

Figure S1: Linkage disequilibrium test between rs2472493 (Site1), rs7636836 (Site 2) and rs61275591 (Site3) using SHEsis online version (http://analysis.bio-x.cn/).
